# Fast CSF MRI for brain segmentation; Cross-validation by comparison with 3D T_1_-based brain segmentation methods

**DOI:** 10.1371/journal.pone.0196119

**Published:** 2018-04-19

**Authors:** Lisa A. van der Kleij, Jeroen de Bresser, Jeroen Hendrikse, Jeroen C. W. Siero, Esben T. Petersen, Jill B. De Vis

**Affiliations:** 1 Department of Radiology, University Medical Center Utrecht, Utrecht, The Netherlands; 2 Spinoza Center for Neuroimaging, Amsterdam, The Netherlands; 3 Danish Research Center for Magnetic Resonance, Center for Functional and Diagnostic Imaging and Research, Copenhagen University Hospital Hvidovre, Hvidovre, Denmark; 4 Center for Magnetic Resonance, DTU Elektro, Technical University of Denmark, Kgs Lyngby, Denmark; 5 National Institute of Neurological Disorders and Stroke, National Institutes of Health, Bethesda, Maryland, United States of America; King’s College London, UNITED KINGDOM

## Abstract

**Objective:**

In previous work we have developed a fast sequence that focusses on cerebrospinal fluid (CSF) based on the long T_2_ of CSF. By processing the data obtained with this CSF MRI sequence, brain parenchymal volume (BPV) and intracranial volume (ICV) can be automatically obtained. The aim of this study was to assess the precision of the BPV and ICV measurements of the CSF MRI sequence and to validate the CSF MRI sequence by comparison with 3D T_1_-based brain segmentation methods.

**Materials and methods:**

Ten healthy volunteers (2 females; median age 28 years) were scanned (3T MRI) twice with repositioning in between. The scan protocol consisted of a low resolution (LR) CSF sequence (0:57min), a high resolution (HR) CSF sequence (3:21min) and a 3D T_1_-weighted sequence (6:47min). Data of the HR 3D-T_1_-weighted images were downsampled to obtain LR T_1_-weighted images (reconstructed imaging time: 1:59 min). Data of the CSF MRI sequences was automatically segmented using in-house software. The 3D T_1_-weighted images were segmented using FSL (5.0), SPM12 and FreeSurfer (5.3.0).

**Results:**

The mean absolute differences for BPV and ICV between the first and second scan for CSF LR (BPV/ICV: 12±9/7±4cc) and CSF HR (5±5/4±2cc) were comparable to FSL HR (9±11/19±23cc), FSL LR (7±4, 6±5cc), FreeSurfer HR (5±3/14±8cc), FreeSurfer LR (9±8, 12±10cc), and SPM HR (5±3/4±7cc), and SPM LR (5±4, 5±3cc). The correlation between the measured volumes of the CSF sequences and that measured by FSL, FreeSurfer and SPM HR and LR was very good (all Pearson’s correlation coefficients >0.83, R^2^ .67–.97). The results from the downsampled data and the high-resolution data were similar.

**Conclusion:**

Both CSF MRI sequences have a precision comparable to, and a very good correlation with established 3D T_1_-based automated segmentations methods for the segmentation of BPV and ICV. However, the short imaging time of the fast CSF MRI sequence is superior to the 3D T_1_ sequence on which segmentation with established methods is performed.

## Introduction

Brain volume measurements on magnetic resonance (MR) images is often used as an etiological and prognostic biomarker [1, 2]. Alzheimer’s disease is a well-known example of a disease characterized by progressive atrophy over the years [3]. Brain volumetrics can also help to monitor the effect of multiple sclerosis treatment [4]. Although qualitative assessment of brain volume is still often used, especially in clinical practice, automatic brain volume measurements have the benefit of providing precise, quantitative brain volume estimates. Widespread use of brain volume measurements in clinical research and clinical practice is hindered by the required 3D T_1_-weighted MRI sequence as it has a long scan time (typically >6min). In addition, the long scan time makes it susceptible to motion artefacts. The 3D T_1_-weighted sequence is aimed at T_1_ contrast, which gives good contrast between gray matter and white matter while CSF appears dark on the images. Recently, we discovered that data of a fast (<1-3min) cerebrospinal fluid (CSF) MRI sequence [5] can be processed to enable brain volume measurements [6]. This short scan time of the CSF sequence is a considerable advantage over the 3D T_1_-weighted sequence and this facilitates a more widespread use of brain volume measurements [6]. The CSF MRI sequence is a 2D sequence with a multislice EPI readout that enables mapping of the T_1_ and T_2_ of CSF. The CSF MRI sequence relies on the longer transverse (T_2_) and longitudinal (T_1_) relaxation rate of the CSF compared to surrounding tissues and allows segmentation of the CSF and brain parenchymal volume (BPV). The obtained images with the CSF MRI sequence show no contrast between gray matter and white matter, and a good contrast between CSF and brain parenchyma. In a previous study, we showed a good correlation between the CSF MRI sequence and qualitative brain atrophy scores, and a good correlation with brain parenchymal volume (BPV) measured by FMRIB Software Library (FSL) in a simple feasibility study [6]. However, the precision of our proposed CSF MRI brain segmentation method has not been assessed nor has it been validated against other 3D T_1_-weighted methods (such as FSL, FreeSurfer and SPM). The aim of this study was to assess the precision of the BPV and intracranial volume (ICV) measurements of the CSF MRI sequence and to validate the CSF MRI sequence by comparison with 3D T_1_-based brain segmentation methods.

## Material and methods

### Participants

For this study ten healthy volunteers (2 females, 8 males) were recruited with a median age of 28 years (range 24–41). Participants were recruited between 2-9-2015 and 22-9-2015, and all participants completed the scan protocol. Recruitment was performed on the university campus. Subjects who demonstrated interest in participating in MR research were approached and screened for MR eligibility. Of the 10 subjects who were approached, all subjects were MRI eligible. This study was approved by the Medical Ethical Committee of the University Medical Center Utrecht under protocol number NL39070.041.11. All participants provided written informed consent.

### MR imaging and data processing

MR imaging was performed on a 3 Tesla Philips Achieva System (Philips, Best, The Netherlands) with a quadrature body coil as transmitter and an 8-channel head coil as a signal receiver. The MRI scan protocol consisted of the following sequences: a low resolution (LR) and a high resolution (HR) CSF MRI sequence, and a high resolution 3D T_1_-weighted sequence. To assess the precision, the scan protocol was performed twice with repositioning of the subjects in between both scans (scan 1 and scan 2). The data of the 3D T_1_-weighted sequence was also downsampled to the voxel size of the HR CSF MRI sequence.

#### CSF MRI sequence

**Fig 1:**
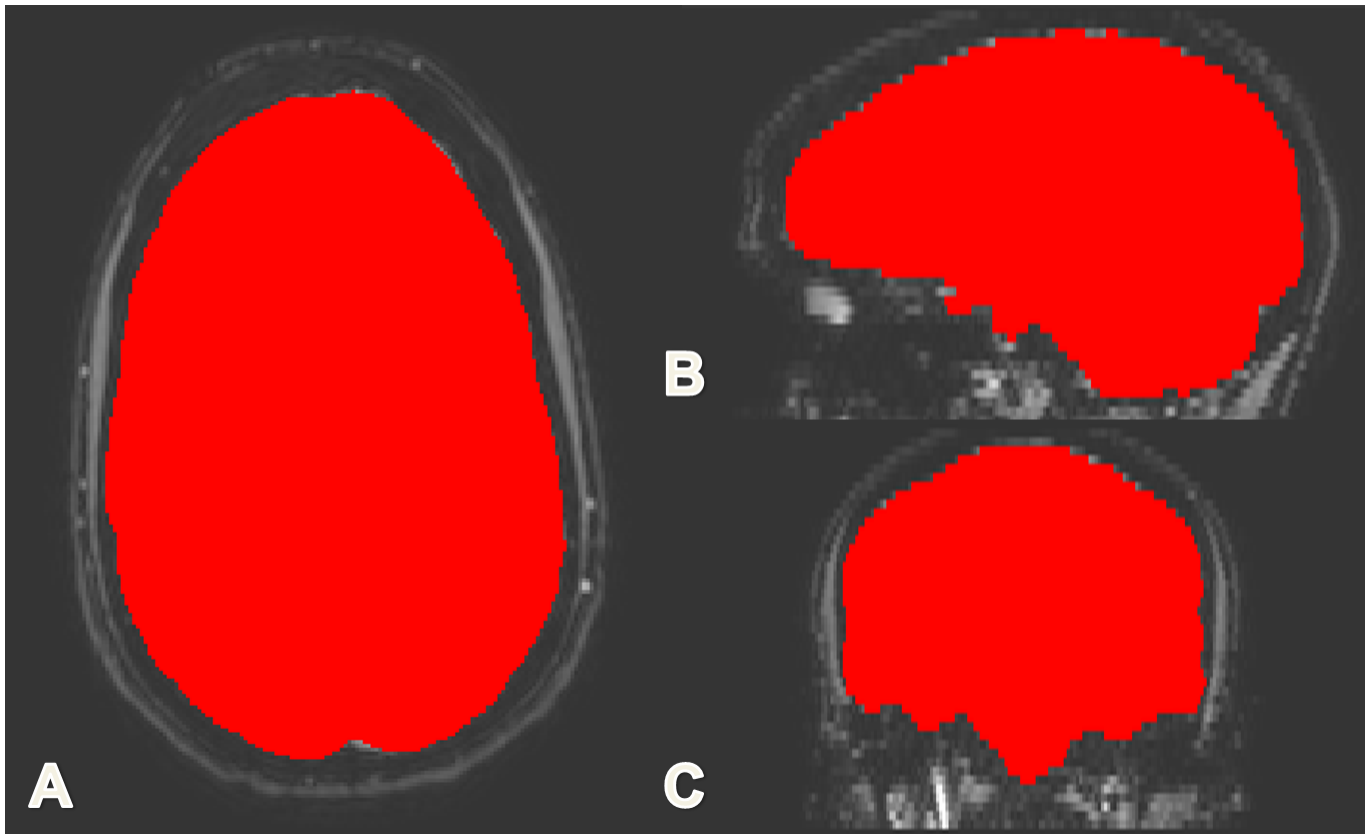
The CSF MRI sequence. After presaturation and T_s_ the signal strength depends on T_1_ decay to ensure an equal magnetization at the start of each MLEV-T_2_ preparation. The MLEV-T_2_ preparation with a varying amount of refocusing pulses allows the mapping of T_2_ decay. Variation of time T_s_ in combination with a fixed number of refocusing pulses allow the mapping of T_1_ decay. A short T_s_ is used to determine signal decay. Multi-slice echo planar imaging is used for the readout at each effective echo time.

As described earlier (for details, see supplemental material in [6], the CSF MRI sequence consists of three parts: a magnetization reset (global non-selective saturation pulse followed by a time delay of 6000ms), a T_2_-preparation, and a readout part (multi-slice echo planar imaging (EPI) readout) [5]. For the T_2_-preparation part, a Malcolm Levitt’s CPD (MLEV) T_2_-preparation scheme was used with a τ_CPMG_ of 70 ms and 0, 4, 8 and 16 refocusing pulses resulting in effective TEs (eTEs) of 0, 280, 560, and 1120 ms (Fig 1). To compensate for the offset caused by different acquisition times in the multi-slice EPI readout, the different eTE encodings were acquired both with and without an inversion pulse, which was added right after the T_2_-preparation module. The final TR of the sequence was 6261 ms. The scan parameters of the low/high resolution CSF MRI sequence were: scan matrix 80x80/240x240, FOV 240x240x161 mm^3^/240x240x161 mm^3^, voxel size 3.0x3.0x7.0 mm^3^/1.0x1.0x3.5 mm^3^, SENSE factor 2.5/2.5, number of slices 23/46, scan time 0:57 min/3:21 min.

Data of the CSF MRI sequences was fully automatically processed using IDL 6.1 for Windows (ITT Visual Information Solutions, Boulder, CO, U.S.A.).

First, signal decay curves were obtained for each voxel from which a voxelwise estimation of the T_2_ of the CSF was performed using the following formula:

$$\begin{array}{l} S\left(eTE,T_{a}\right)-S\left(T_{a}\right)=A\cdot e^{\frac{-eTE}{T_{2,CSF}}}\\ A=M_{0}\cdot \left(1-e^{\frac{-T_{s}}{T_{1,CSF}}}\right)\cdot e^{\frac{-T_{a}}{T_{1,CSF}}} \end{array}$$ (1)

where S(T_a_) is the signal recovery at time T_a_, the constant A is the recovered magnetization at time T_s_ after the non-selective saturation pulse, T_1,CSF_ and T_2,CSF_ are the longitudinal and transverse relaxation of the CSF. Second, the M_0_ of the voxels were obtained by extrapolating the T_2_ of the CSF to the intersect at eTE = 0 ms. From this, the actual volume fraction of CSF (CSF_vf_) in a voxel is obtained by relating the M_0_ of a voxel to the M_0_ of reference voxels. Only last 3 echo times are used to extrapolate the M_0_ of CSF in order to exclude parenchymal tissue. The reference voxels free of partial volume are automatically selected based on a histogram analysis of signal intensities. The CSF volume was based on the CSF M_0_ map which was smoothed with a 7mm Gaussian filter. The signal value at 99.5% of the nonzero histogram was used to determine the reference voxels filled with CSF. The upper 0.5% of values were discarded to avoid artificially high reference values caused by noise. Third, total CSF volume (Vcsf) was obtained by multiplying the volume fraction by the voxel size (mm^3^).

$$CSF_{VF}=\frac{S\left(eTE=0\right)}{S_{ref}\left(eTE=0\right)}$$ (2)

**Fig 2:**
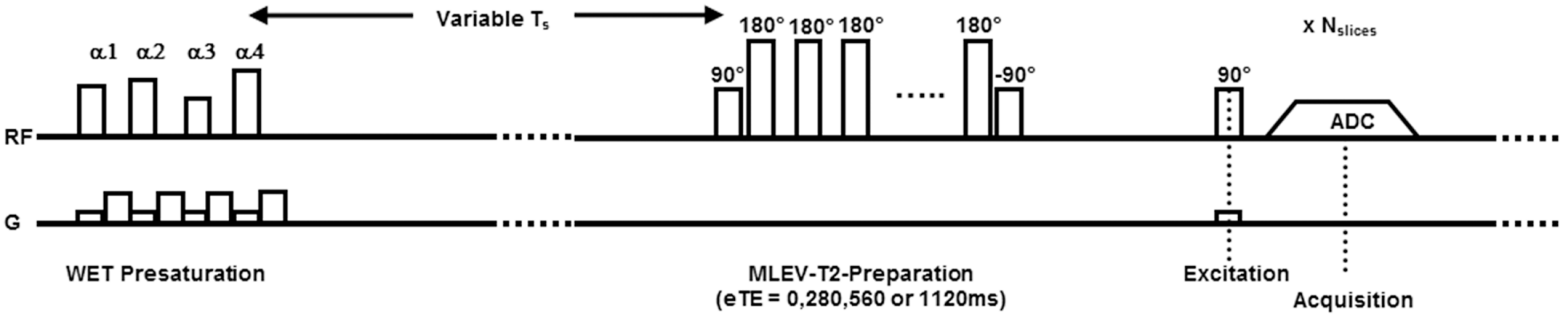
The ICV derived from the CSF MRI sequence. Example of the ICV in an axial (A), saggital (B) and coronal (C) slice. The mask resulting from the BET application to the CSF data is shown in red. The raw CSF data is shown in gray-scale in the background.

Fourth, based on the first acquired echo (eTE = 0) of the CSF MRI sequence which includes both parenchymal and CSF signal, the intracranial volume (ICV) was measured. A conservative brain extraction (BET) mask from FSL [7] was applied to the CSF map to remove the skull and eyes. The CSF map and skull stripped (BET) eTE = 0 images were added and subsequently smoothed with a Gaussian filter of 3mm. Then, the threshold of any signal above 3% of the resulting histogram was assigned to the ICV. The assignment of voxels to the ICV was binary (Fig 2). The brain parenchymal volume was calculated from the difference between the ICV and total CSF volume.

The postprocessing time was 1 minute for the low resolution CSF MRI sequence (CSF LR), and it was 25 minutes for the high resolution CSF MRI sequence (CSF HR).

#### 3D T1-weighted sequence and 3D T1-based segmentation methods

MR imaging parameters of the HR 3D-T_1_-weighted MRI sequence were; matrix size 256x256, FOV 232x256x192, voxel size 1.0x1.0x1.0 mm^3^, TR = 8187ms, TE = 4.5ms, scan time 6:47 min. The data were downsampled to LR 3D-T_1_-weighted images with a resolution similar to the CSF HR MRI sequence through registration to the CSF HR images: voxel size 1.0x1.0x3.5 mm^3^, scan time 1:59 min (MeVis Medical Solutions AG, Bremen, Germany, version 2.7). The 3D T_1_-weighted images were used for brain segmentation by FMRIB Software Library 5.0 (FSL), FreeSurfer (5.3.0) and Statistical Parametric Mapping 12 (SPM).

#### FSL

FSL is software created by the Analysis Group, FMBRIB, Oxford, UK. In FSL, Structural Image Evaluation using Normalization of Atrophy Cross-sectional (SIENAX) was used to obtain brain volumes normalized for skull size [8, 9]. The—B option was used for bias field correction and neck clean up [10]. The brain extraction tool was used to strip non-brain tissue and the f-parameter was set at 0.2, because the best overall results were obtained with this setting as tested by visual inspection. In SIENAX, both the skull and extracted brain were registered to standard space to allow the calculation of (peripheral) CSF volume and the removal of structures such as the eyes with a probabilistic map [8]. The gray matter and white matter were taken together to obtain the BPV. The CSF was added to the BPV to obtain the ICV.

#### FreeSurfer

FreeSurfer is open source software developed by the Athinoula A. Martinos Center for Biomedical Imaging, Harvard-MIT, Boston. The volume-based stream consists of five steps, including registration to standard space and skull stripping [11]. In Freesurfer, each voxel is assigned to a certain structure. The probability of the voxel belonging to a particular structure is modulated by the neighboring voxels, because they are expected to belong to the same structure [10]. Automated brain segmentation in FreeSurfer (surfer.nmr.mgh.harvard.edu) was performed using the standard settings (recon-all—all). BPV and ICV were obtained from the stats file that becomes available after segmentation (asegstats2table).

#### SPM

SPM12 is developed by the Wellcome Department of Cognitive Neurology, Institute of Neurology, Queen Square, London [12]. Neck slices were removed in FSL to preprocess the images. Brain volumes were obtained from the pre-processed images with the ‘Segment’ function (http://www.fil.ion.ucl.ac.uk/spm). This function uses the Unified Segmentation algorithm, which combines image registration, tissue classification and bias correction [13]. As such, both the signal intensity of the voxel and the location through registration with tissue probability maps determines the probability of a voxel belonging to a certain tissue class. The recommended default settings were used. As such, the number of Gaussians for white matter and gray matter were set from one to two. The ICV and BPV were calculation from the obtained gray matter, white matter and CSF volumes.

### Statistical analysis

Statistical analysis was carried out with R (R Foundation for Statistical Computing, Vienna, Austria).

#### Precision

For both BPV and ICV the precision was calculated by the mean (absolute) differences between the measurements of scan 1 and scan 2 for the CSF sequences and the 3D T_1_-based methods (FSL, FreeSurfer, SPM). The mean difference between the results of scan 1 and scan 2 reflects whether a systematic bias exists in the test-retest measurements. The mean absolute difference reflects the total test-retest volume error between the results of scan 1 and 2. Wilcoxon signed rank tests were used to evaluate whether there was a significant difference between the test-retest measurements. Bland-Altman plots were used to visualize these differences [14]. Precision was compared between the CSF sequences and the 3D T_1_-based methods.

#### Comparison between methods

The absolute volumes of BPV and ICV between the CSF sequences and the 3D T_1_-based methods (FSL, FreeSurfer, SPM) were evaluated for significant differences (Wilcoxon signed rank). To test for correlation between the CSF sequences and the 3D T_1_-based methods, Pearson’s correlation coefficients were calculated. Bland-Altman plots comparing the volumes obtained with the CSF sequence with those of the 3D T_1_-based methods were used to visualize differences. The results from the first scan were used for the Bland-Altman plots. The degree of variance was calculated (R^2^) with 90% confidence intervals (CI).

## Results

**Fig 3:**
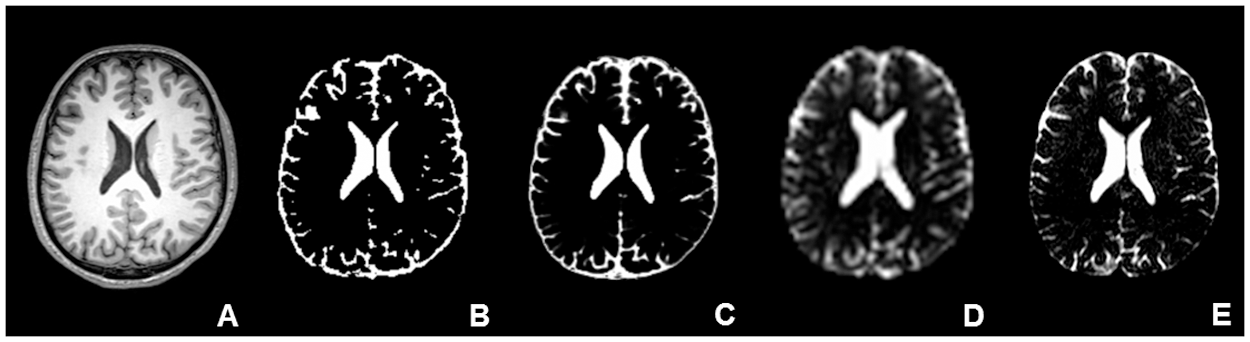
All CSF measurements for all methods in each subject. One slice from subject 8 from A) the HR T_1_-weighted image (downsampled images have the same in-plane resolution); B) 3D T_1_-weighted FSL HR; C) 3D T_1_-weighted SPM HR; D) postprocessed CSF LR; E) postprocessed CSF HR. No image is presented for FreeSurfer, because FreeSurfer uses the determinant of the transformation matrix to estimate ICV rather than distinguishing the skull from peripheral CSF.

All segmentations of all methods were considered of good quality (see Fig 3 for an example).

### BPV

#### Precision

**Fig 4:**
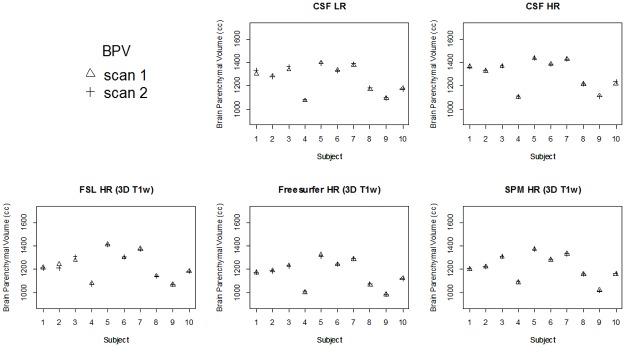
All BPV measurements for all methods in each subject. CSF LR = CSF low resolution MRI scan; CSF HR = CSF high resolution MRI scan.

**Fig 5:**
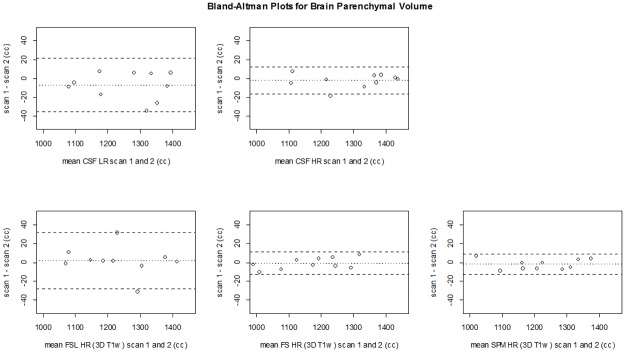
Bland Altman plots for precision of BPV per method. CSF LR, CSF HR (top row), FSL, FreeSurfer and SPM (bottom row). The dashed lines represent the limits of agreement (mean difference ±1.96*SD of the differences). CSF LR = CSF low resolution MRI scan; CSF HR = CSF high resolution MRI scan; FS = FreeSurfer.

Fig 4 shows all BPV measurements for all methods in each subject. Table 1 shows the mean BPV for the first and second scan and the mean (absolute) differences between measurements on these scans for each of the methods in cc. The mean difference is the average of all test-retest differences to indicate whether there is a bias between the two measurements. The mean absolute difference is the average of the absolute test-retest differences, and it provides a measure of precision as it contains the cumulative error. The BPV measured on the first and second scan were not significantly different within each method (p>0.05). The mean differences for BPV between the first and second scan for the CSF LR (-7±14 cc) and CSF HR (2±7 cc) sequences were in the range of those for FSL HR (-2±15 cc), FSL LR (4±7 cc), FreeSurfer HR (1±6 cc), FreeSurfer LR (1±12 cc), SPM HR (2±5 cc), and SPM LR (-2±6 cc). The mean absolute differences for BPV between scans were comparable for the CSF HR sequence (5±5 cc) and FSL HR (9±11 cc), FSL LR (7±4 cc), FreeSurfer HR (5±2 cc), FreeSurfer LR (9±8 cc), SPM HR (5±3 cc), and SPM LR (5±4 cc) but the CSF LR (12±9 cc) showed somewhat larger mean absolute differences than FreeSurfer HR and SPM HR and LR. The Bland-Altman plots comparing the BPV obtained at the first scan versus that of the second scan for the CSF MRI sequence and the HR 3D T_1_-based methods are shown in Fig 5. The Bland-Altman plots from the downsampled 3D T_1_ sequence are shown in S2 Fig. The mean in the Bland-Altman plots should be close to 0, indicating that there is a small absolute difference between scan 1 and scan 2 within methods. The mean was close to 0 for all methods. The limits of agreement in the Bland-Altman plots should be narrow, indicating a good precision. The limits of agreement were most narrow for SPM HR (-13–9 cc) and FreeSurfer HR (-13–11 cc), and SPM LR (-11–15).

**Table 1:** Precision of BPV and ICV for CSF LR, CSF HR and automated segmentation methods with downsampled data.

	CSF LR (cc)	CSF HR (cc)	FSL HR (cc)	FreeSurfer HR (cc)	SPM HR (cc)	FSL LR^a^ (cc)	FreeSurfer LR^a^ (cc)	SPM LR^a^ (cc)
**BPV**								
*Scan 1*	1255±111	1296±117	1231±109	1164±109	1216±105	1257±108	1172±110	1244±116
*Scan 2*	1262±112	1298±116	1229±112	1165±106	1218±105	1261±104	1173±101	1242±118
*Mean difference between scans*	-7.0±13.9	1.9±7.0	-2.2±14.5	0.9±5.7	2.0±5.3	3.8±7.3	1.2±11.7	-1.9±6.2
*Mean absolute difference between scans*	12.4±9.4	5.3±4.9	9.2±11.4	5.1±2.6	5.0±2.7	7.2±4.1	9.0±7.5	5.0±4.1
**ICV**								
*Scan 1*	1481±130	1499±137	1598±136	1555±251	1513±135	1690±145	1707±151	1497±123
*Scan 2*	1481±129	1500±136	1595±140	1567±253	1515±140	1691±141	1714±152	1494±122
*Mean difference between scans*	0.0±7.8	1.2±4.3	-3.6±29.5	11.8±10.4	2.4±7.4	0.9±7.6	6.6±14.1	-3.4±4.5
*Mean absolute difference between scans*	6.9±3.7	3.9±2.1	18.5±23.3	13.5±8.2	4.3±6.5	6.0±4.7	11.8±10.2	5.0±2.7

Value s represent means±SD in cc. CSF LR = CSF low resolution MRI scan; CSF HR = CSF high resolution MRI scan.

^a^The automated segmentations were performed on downsampled (LR) T_1_-weighted scans with a resolution equal to the CSF HR image: 1x1x3.5 mm^3^.

#### Comparison between methods

**Fig 6:**
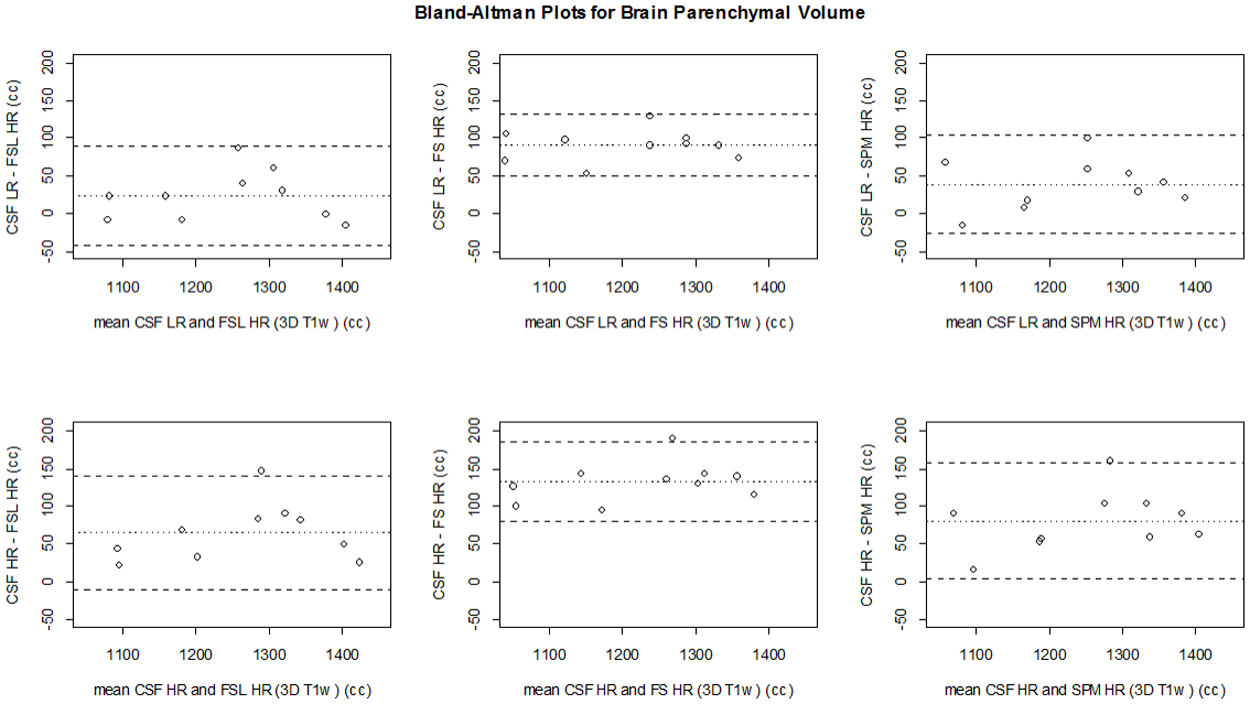
Bland Altman plots for the comparison of BPV between the CSF MRI sequences and the 3D T_1_-based brain segmentation methods. The dashed lines represent the limits of agreement (mean difference ±1.96*SD of the differences). CSF LR = CSF low resolution MRI scan; CSF HR = CSF high resolution MRI scan; FS = FreeSurfer.

The CSF sequences measured significantly higher BPV compared to the HR 3D T_1_-based methods (FSL HR, FreeSurfer HR, SPM HR), except for CSF LR versus FSL HR (Table 2). The CSF HR sequence measured a significantly higher BPV than FreeSurfer LR (S1 Table). However, both the CSF LR and CSF HR measurements of BPV showed a very good correlation with the 3D T_1_-based segmentation methods (all Pearson’s correlation coefficients >0.93; Table 2, S1 Table). The Bland-Altman plots comparing the BPV obtained with the CSF sequences with those of the 3D T_1_-based methods are shown in Fig 6. The mean in the Bland-Altman plots should be close to 0, indicating that there is a small absolute difference between the CSF sequences and the 3D T_1_-based methods. In the Bland-Altman plots, the mean was closest to 0 for CSF LR/FSL LR (-2 cc), CSF LR/SPM LR (11 cc), and CSF LR/FSL HR (24 cc), CSF HR/FSL LR (39 cc) and CSF LR/SPM HR (39 cc), (Fig 6). The limits of agreement in the Bland-Altman plots should be narrow, indicating a small variation in measurements between the CSF sequences and the 3D T_1_-based methods. The limits of agreement were the most narrow for CSF LR/FreeSurfer HR (49–133 cc), CSF LR/FreeSurfer LR (35–130), CSF HR/FreeSurfer HR (79–185 cc), and CSF LR/FSL LR (-61–56) (Fig 6). The degree of variance was comparable between all measures (R^2^ 0.88–0.97, Table 2).

**Table 2:** Correlation between the CSF MRI sequences and the HR 3D T_1_-based brain segmentation methods.

	Scan 1			Scan 2		
Correlation tested	Mean Δvolume (cc)	Pearson’s Correlation Coefficient	R^2^ (90% CI)	Mean Δvolume (cc)	Pearson’s Correlation Coefficient	R^2^ (90% CI)
**CSF LR BPV**						
*FSL HR*	24±32	.98	.92 (.77-.97)	33±40*	.94	.88 (.64-.96)
*FreeSurfer HR*	91±20*	.96	.97 (.88-.99)	97±31*	.96	.92 (.75-.97)
*SPM HR*	39±32*	.96	.92 (.75-.96)	44±38*	.94	.88 (.60-.96)
**CSF HR BPV**						
*FSL HR*	65±37*	.95	.90 (.70-.97)	69±37*	.95	.90 (.75-.97)
*FreeSurfer HR*	132±25*	.98	.95 (.82-.99)	133±22*	.98	.97 (.88-.99)
*SPM HR*	80±37*	.95	.90 (.65-.96)	80±35*	.95	.91 (.72-.96)
**FSL HR BPV**						
*FreeSurfer HR*	67±18	.99	.97 (.95-.99)	64±20	.98	.97 (.92-.99)
*SPM HR*	15±24	.98	.95 (.85-.98)	11±26	.97	.95 (.86-.97)
**Freesurfer HR BPV**						
*SPM*	-52±21	.98	.96 (.91-.98)	-53±21	.98	.96 (.91-.98)
**CSF LR ICV**						
*FSL HR*	-117±52*	.93	.86 (.44-.99)	-114±54*	.92	.85 (.47-.99)
*FreeSurfer HR*	-74±153	.86	.75 (.49-.90)	-86±157	.86	.74 (.52-.88)
*SPM HR*	-32±50	.92	.86 (.48-.94)	-34±56	.92	.84 (.42-.92)
**CSF HR ICV**						
*FSL HR*	-99±49*	.93	.87 (.49-.98)	-95±50*	.94	.87(.53-.98)
*FreeSurfer HR*	-56±155	.84	.70 (.38-.88)	-67±159	.83	.69 (.40-.87)
*SPM HR*	-14±48	.94	.88 (.53-.93)	-15±52	.93	.86 (.53-.92)
**FSL HR ICV**						
*FreeSurfer HR*	43±152	.86	.73 (.49-.87)	28±151	.86	.74 (.48-.88)
*SPM HR*	85±39	.96	.92 (.75-.97)	79±42	.95	.91 (.73-.96)
**Freesurfer HR ICV**						
*SPM HR*	42±158	.83	.69 (.41-.86)	52±160	.82	.67 (.33-.86)

CSF LR = CSF low resolution MRI scan; CSF HR = CSF high resolution MRI scan

*p < 0.05.

### ICV

#### Precision

**Fig 7:**
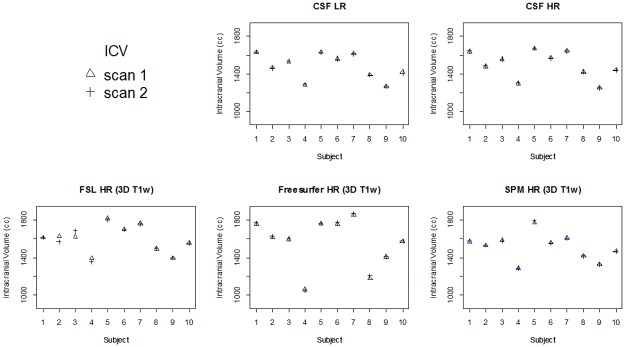
All ICV measurements for all methods in each subject. CSF LR = CSF low resolution MRI scan; CSF HR = CSF high resolution MRI scan.

**Fig 8:**
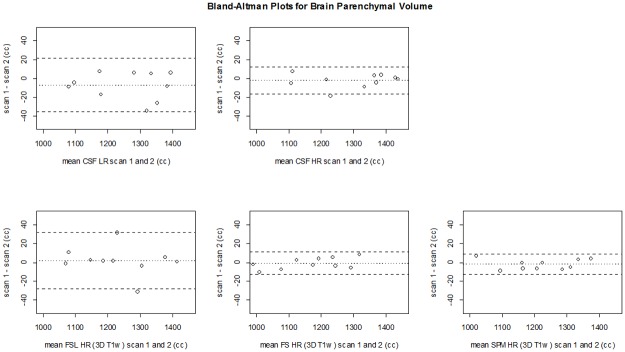
Bland Altman plots for precision of ICV per method. CSF LR, CSF HR (top row), FSL, FreeSurfer and SPM (bottom row). The dashed lines represent the limits of agreement (mean difference ±1.96*SD of the differences). CSF LR = CSF low resolution MRI scan; CSF HR = CSF high resolution MRI scan; FS = FreeSurfer.

Fig 7 shows all ICV measurements for all methods in each subject. Table 1 shows the mean ICV for the first and second scan, and the mean (absolute) differences between measurements on these scans for each of the methods. The ICVs measured on the first and second scan were not significantly different within each method (p>0.05), except for SPM LR (p<0.05). The mean differences for ICV between scans of the CSF LR (0±8 cc) and CSF HR (1±4 cc) sequences were smaller than for the 3D T_1_-based brain segmentation methods (Table 1). For ICV, the mean absolute differences between scans of the CSF LR (7±4 cc) and CSF HR (4±2 cc) sequences were small and within the range of FSL HR (19±23 cc), FSL LR (6±5 cc), FreeSurfer HR (14±8 cc), FreeSurfer LR (12±10), SPM HR (4±7 cc), and SPM LR (5±3 cc). The Bland-Altman plots comparing the ICV obtained at the first scan versus that of the second scan for all methods are shown in Fig 8. In the Bland-Altman plots, only FreeSurfer HR showed a mean that was somewhat further away from 0. The limits of agreement were most narrow for CSF HR (-10–8 cc), and SPM LR (-6–13 cc).

#### Comparison between methods

**Fig 9:**
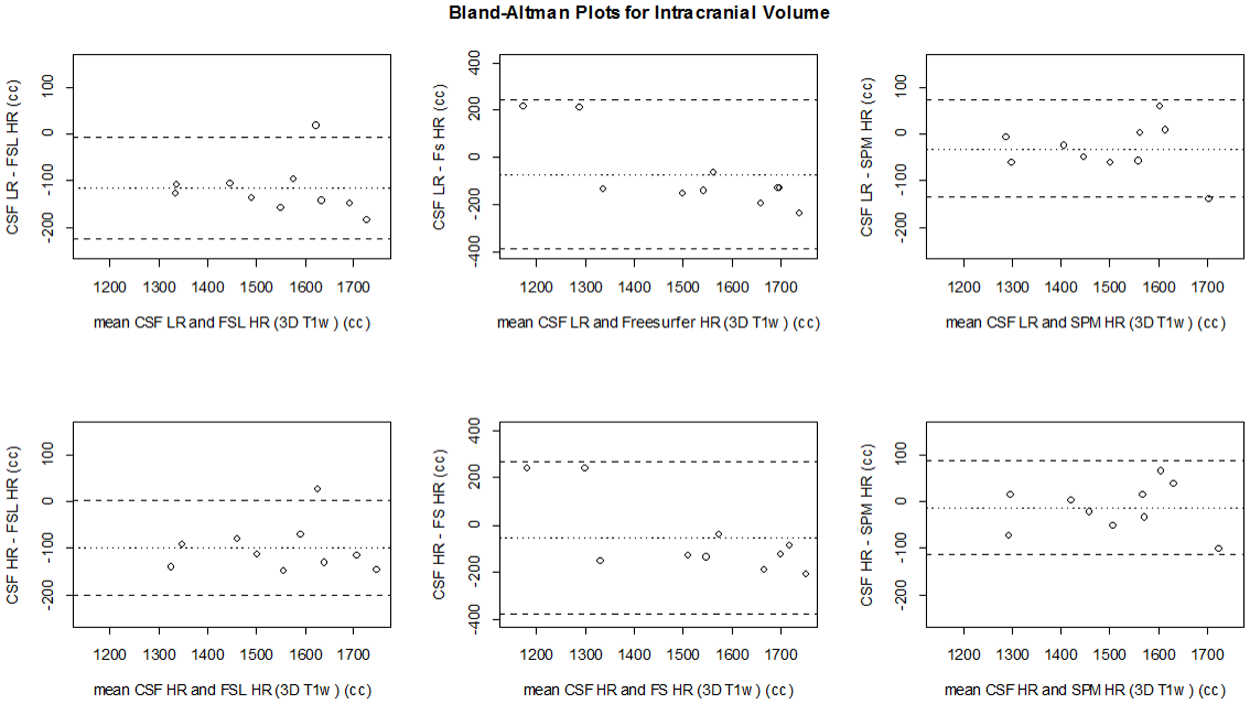
Bland Altman plots for the comparison of ICV between the CSF MRI sequences and the 3D T_1_-based brain segmentation methods. The dashed lines represent the limits of agreement (mean difference ±1.96*SD of the differences). CSF LR = CSF low resolution MRI scan; CSF HR = CSF high resolution MRI scan; FS = FreeSurfer.

The CSF sequences measured somewhat lower ICV compared to the HR 3D T_1_-based methods (FSL, FreeSurfer, SPM), but this was only significantly different for the CSF sequences versus FSL (p<0.05). The CSF sequences measured a lower ICV than FSL LR and FreeSurfer LR (p<0.05). There was a good to very good correlation for ICV measurements between the CSF sequences and the 3D T_1_-based methods (all Pearson’s correlation coefficients >0.83, Table 2, S1 Table). The Bland-Altman plots comparing the ICV obtained with the CSF sequences with those of the 3D T_1_-based methods are shown in Fig 9 and in S6 Fig. In the Bland-Altman plots, the mean was closest to 0 for CSF HR/SPM LR (1 cc), CSF HR/SPM (-14 cc), CSF LR/SPM LR (-17 cc), and CSF LR/SPM HR (-32 cc). The limits of agreement were the most narrow for CSF HR/FreeSurfer LR (-264 –-153 cc), CSF LR/FreeSurfer LR (-296 –-157 cc), CSF HR/FSL LR (-276 –-106 cc), CSF LR/FSL LR (-298 –-120 cc), and CSF LR/SPM LR (-109–76 cc). The degree of variance was comparable between all measures (R^2^ 0.84–0.92), except for FreeSurfer (R^2^ 0.67–0.75, Table 2).

## Discussion

This study demonstrates that the proposed CSF MRI sequences perform similarly in terms of precision (test-retest) and obtained volumes as conventional 3D T_1_-weighted MRI sequences in the assessment of BPV and ICV, but with a much shorter scan time. The automated segmentation methods performed similarly for high resolution and downsampled 3D T_1_-weighted data for BPV and ICV. Furthermore, an excellent correlation was found between the BPVs and ICVs obtained with the CSF MRI sequences and those obtained with the 3D T_1_-weighted MRI sequences using FSL, FreeSurfer and SPM.

The precision and performance of commonly used MRI-based segmentation methods varies across different methods and also varies dependent on the application [15–17]. The observed precision of our CSF MRI sequences were in line with these previous studies of commonly used brain segmentation methods. As was expected, the CSF HR sequence showed a slightly higher precision than the CSF LR sequence. The CSF sequences even showed a markedly higher precision for ICV than FSL in this study. This is caused by the limited contrast on 3D T_1_-weighted images between the tabula interna of the skull and the dura mater on one side, and the CSF on the other side [18]. As a result, the ICV that is calculated on 3D T_1_-weighted images can be troublesome and several segmentation methods show difficulties in calculating the ICV [18–20]. Especially FreeSurfer has been shown earlier to perform less well than other methods for ICV [15, 16]. In this study, the mean absolute difference for ICV is two-to-four times lower for the two CSF sequences than for FSL and FreeSurfer, indicating a better precision.

The short imaging time of 57 seconds is a major advantage of the CSF LR sequence over 3D T_1_ based methods, because it facilitates an easy addition of this sequence to a scan protocol. The 3D T_1_-weighted sequence used in our study had a scan time of nearly 7 minutes and this scan could thus be avoided when a high-resolution T_1_- weighted scan is not necessary to answer any of the other clinical or research questions. Nowadays, shorter 3D T_1_-weighted sequences that still provide sufficient image quality for most radiology exams are available, and we simulated the precision of such a sequence by downsampling the data of the high resolution T_1_-weighted sequence. The results of this analysis demonstrated that global measures from automated segmentation methods—BPV and ICV—are not sensitive to a somewhat lower scan quality. The precision of our LR CSF sequence was similar to the precision of both the HR and LR T_1_-weighted sequence, but then with a scan time half of the scan time of the LR T_1_-weighted sequence. Another strength of our CSF sequences is the good contrast between the skull and CSF, which is often limited in 3D T_1_-weighted images. This leads to an very good precision of our CSF sequences for ICV. A limitation of our CSF sequences is that there is almost no contrast between the white and gray matter. Consequently, both gray and white matter atrophy and cortical thickness cannot be distinguished. Another limitation of the CSF LR and HR sequences could be that the obtained volumes are not similar to the volumes obtain with SPM, FreeSurfer or FSL. However, this also holds for comparisons across these 3D T1-based segmentation methods [15, 16]. More importantly, we have shown that there is an excellent correlation between the estimated volumes from the CSF sequences and the 3D T1-based methods.

The purpose of this paper was to cross-validate the CSF MRI sequences for volume estimates. Future work could focus on further image processing of the CSF images such that regional brain atrophy can be determined. This would allow detection of different patterns of brain atrophy. This is a useful addition, as several diseases are marked by a characteristic pattern of brain atrophy, for example frontotemporal dementia [21]. The postprocessing time of 1 minute could also be further decreased in future studies to enable an easier implementation in clinical practice. In addition, the CSF sequences could be further validated in cohorts of patients with more brain abnormalities and variations in brain pathology. The CSF sequence might provide information about the CSF. For example, the T_2,_ of CSF may be related to oxygen partial pressure [5, 22].

In conclusion, both CSF MRI sequences have a precision comparable to, and a very good correlation with established 3D T_1_-based automated segmentations methods for segmentation of BPV and ICV. However, the short imaging time of the fast CSF MRI sequence is superior to the 3D T_1_-weighted sequence on which segmentation with established methods is performed.

## Supporting information

S1 Dataset (XLSX)

S1 Fig All BPV measurements for all methods in each subject. CSF LR = CSF low resolution MRI scan; CSF HR = CSF high resolution MRI scan.
(PNG)

S2 Fig Bland Altman plots for precision of BPV per method. CSF LR, CSF HR (top row), FSL LR, FreeSurfer LR, and SPM LR (bottom row). The dashed lines represent the limits of agreement (mean difference ±1.96*SD of the differences). LR = low resolution, downsampled scan. CSF LR = CSF low resolution MRI scan; CSF HR = CSF high resolution MRI scan; FS = FreeSurfer.
(PNG)

S3 Fig Bland Altman plots for the comparison of BPV between the CSF MRI sequences and the downsampled (LR) 3D T_1_-based brain segmentation methods. The dashed lines represent the limits of agreement (mean difference ±1.96*SD of the differences). CSF LR = CSF low resolution MRI scan; CSF HR = CSF high resolution MRI scan; FS = FreeSurfer.
(PNG)

S4 Fig All ICV measurements for all methods in each subject. CSF LR = CSF low resolution MRI scan; CSF HR = CSF high resolution MRI scan.
(PNG)

S5 Fig Bland Altman plots for precision of ICV per method. CSF LR, CSF HR (top row), FSL LR, FreeSurfer LR, and SPM LR (bottom row). CSF LR, CSF HR (top row), FSL LR, FreeSurfer LR, and SPM LR (bottom row). The dashed lines represent the limits of agreement (mean difference ±1.96*SD of the differences). LR = low resolution, downsampled scan. CSF LR = CSF low resolution MRI scan; CSF HR = CSF high resolution MRI scan; FS = FreeSurfer.
(PNG)

S6 Fig Bland Altman plots for the comparison of ICV between the CSF MRI sequences and the downsampled (LR) 3D T_1_-based brain segmentation methods. The dashed lines represent the limits of agreement (mean difference ±1.96*SD of the differences). CSF LR = CSF low resolution MRI scan; CSF HR = CSF high resolution MRI scan; FS = FreeSurfer.
(PNG)

S1 Table Correlation between the CSF MRI sequences and the LR 3D T_1_-basedbrain segmentation methods. (PDF)

S2 Table Table regression parameters for the relationship between the CSF MRI sequences and the HR 3D T1-based brain segmentation methods. (PDF)

S3 Table Table regression parameters for the relationship between the CSF MRI sequences and the LR 3D T1-based brain segmentation methods. (PDF)
