# Supplementary figures and images for: Fast CSF MRI for brain segmentation; Cross-validation by comparison with 3D T1-based brain segmentation methods
Source: PLoS One. 2018 Apr 19;13(4):e0196119. doi: 10.1371/journal.pone.0196119 (PMC5908081; doi:10.1371/journal.pone.0196119)

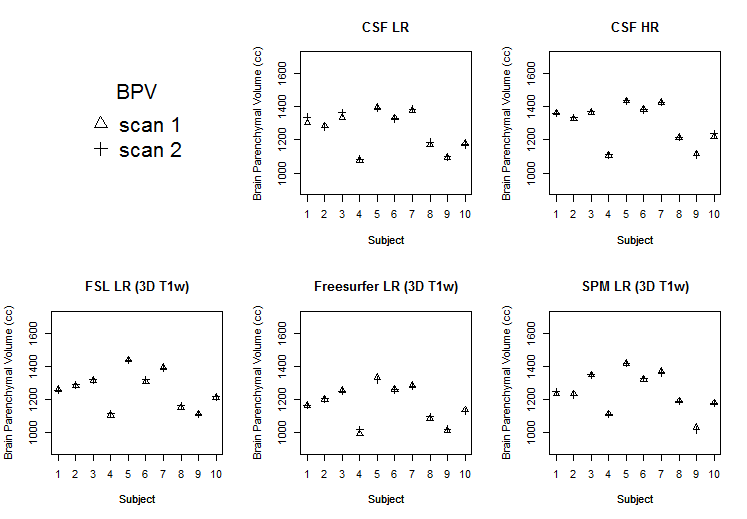

Supplement: S1 Fig — CSF LR = CSF low resolution MRI scan; CSF HR = CSF high resolution MRI scan. (PNG) [file pone.0196119.s002.png]

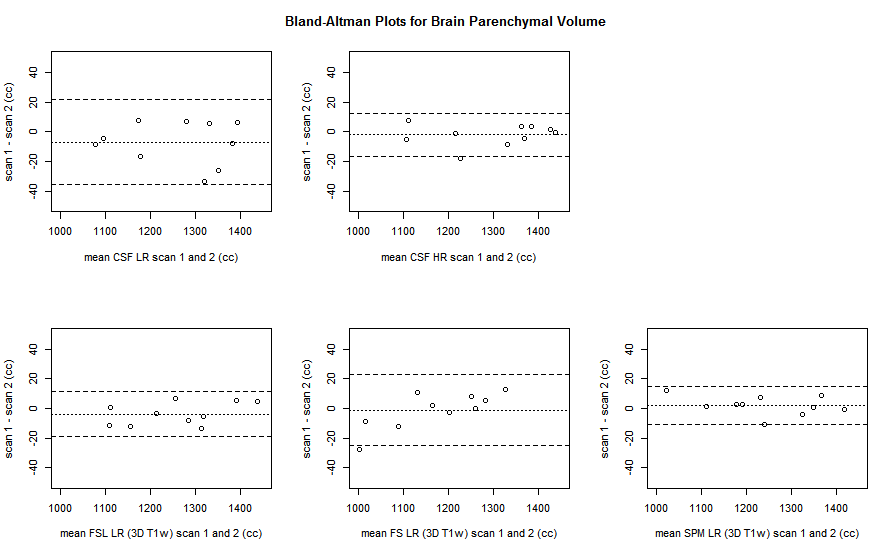

Supplement: S2 Fig — CSF LR, CSF HR (top row), FSL LR, FreeSurfer LR, and SPM LR (bottom row). The dashed lines represent the limits of agreement (mean difference ±1.96*SD of the differences). LR = low resolution, downsampled scan. CSF LR = CSF low resolution MRI scan; CSF HR = CSF high resolution MRI scan; FS = FreeSurfer. (PNG) [file pone.0196119.s003.png]

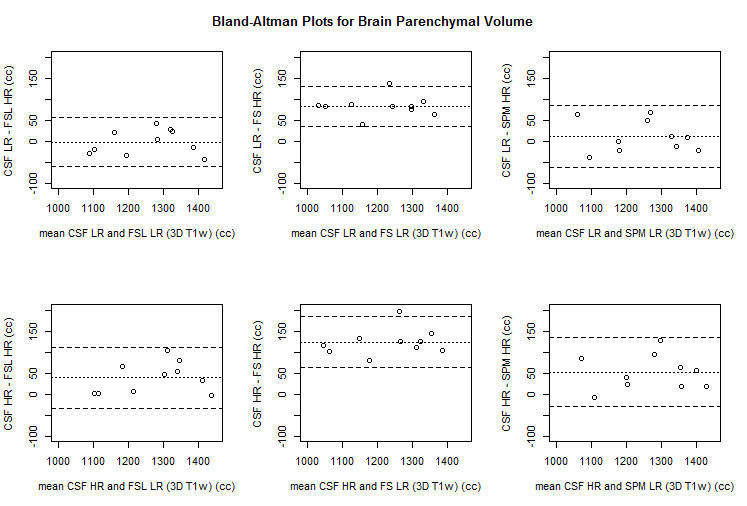

Supplement: S3 Fig — The dashed lines represent the limits of agreement (mean difference ±1.96*SD of the differences). CSF LR = CSF low resolution MRI scan; CSF HR = CSF high resolution MRI scan; FS = FreeSurfer. (PNG) [file pone.0196119.s004.png]

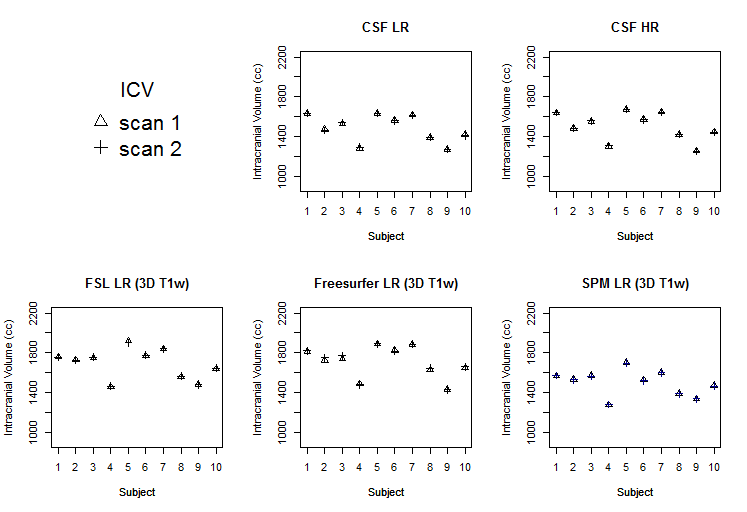

Supplement: S4 Fig — CSF LR = CSF low resolution MRI scan; CSF HR = CSF high resolution MRI scan. (PNG) [file pone.0196119.s005.png]

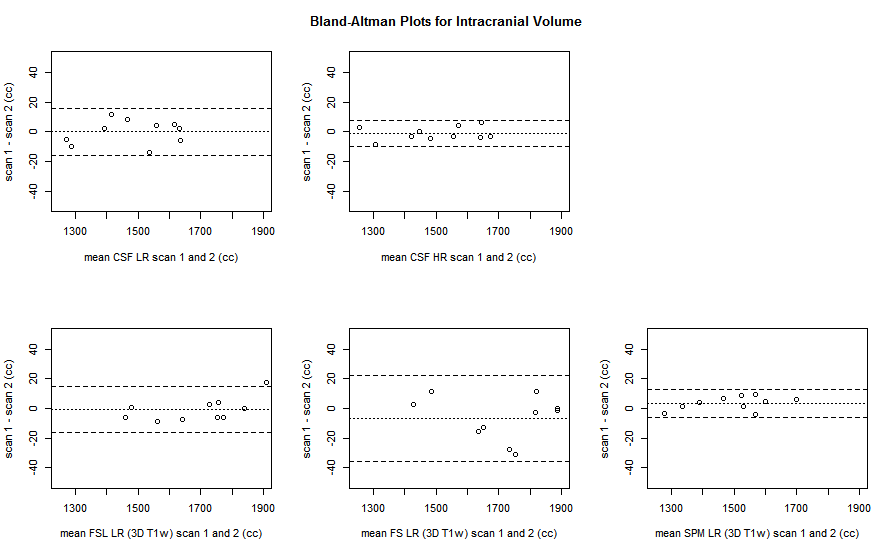

Supplement: S5 Fig — CSF LR, CSF HR (top row), FSL LR, FreeSurfer LR, and SPM LR (bottom row). The dashed lines represent the limits of agreement (mean difference ±1.96*SD of the differences). LR = low resolution, downsampled scan. CSF LR = CSF low resolution MRI scan; CSF HR = CSF high resolution MRI scan; FS = FreeSurfer. (PNG) [file pone.0196119.s006.png]

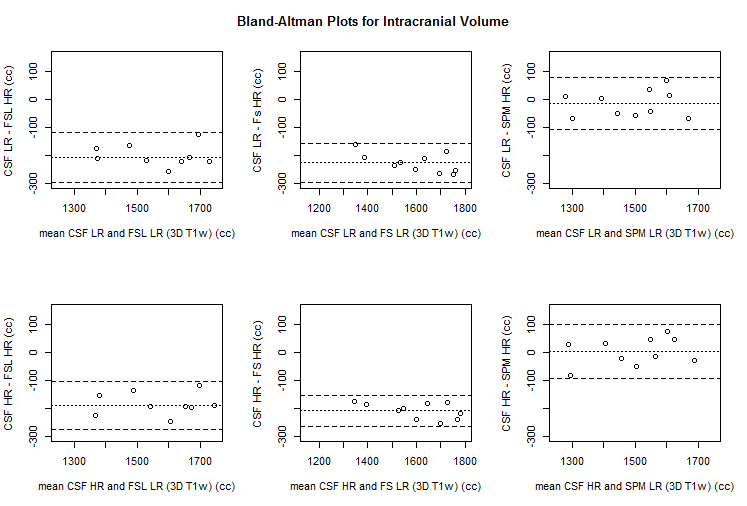

Supplement: S6 Fig — The dashed lines represent the limits of agreement (mean difference ±1.96*SD of the differences). CSF LR = CSF low resolution MRI scan; CSF HR = CSF high resolution MRI scan; FS = FreeSurfer. (PNG) [file pone.0196119.s007.png]
