# Supplementary material for: Fast CSF MRI for brain segmentation; Cross-validation by comparison with 3D T1-based brain segmentation methods
Source: PLoS One. 2018 Apr 19;13(4):e0196119. doi: 10.1371/journal.pone.0196119 (PMC5908081; doi:10.1371/journal.pone.0196119)
Supplement: S1 Table — (PDF) [file pone.0196119.s008.pdf]

	Scan 1			Scan 2		
Correlation tested	Mean Δvolume (cc)	Pearson's Correlation Coefficient	R^2 (90% CI)	Mean Δvolume (cc)	Pearson's Correlation Coefficient	R^2 (90% CI)
**CSF LR BPV**						
FSL LR	-2±28	.97	.93 (.84-.97)	1±39	.94	.88 (.62-.96)
FreeSurfer LR	83±23	.98	.96 (.82-.99)	89±35	.95	.91 (.64-.98)
SPM LR	11±36	.95	.90 (.76-.95)	20±40	.94	.89 (.65-.94)
**CSF HR BPV**						
FSL LR	39±35	.95	.91 (.77-.96)	37±33	.96	.92 (.76 -.97)
FreeSurfer LR	124±30*	.97	.94 (.74-.99)	125±29*	.97	.95 (.79 -.99)
SPM LR	52±40	.94	.89 (.68-.95)	56±38	.95	.90 (.76-.96)
**FSL LR BPV**						
FreeSurfer LR	89±19	.98	.96 (.88-.98)	88±17	.99	.97 (.92-.99)
SPM LR	17±33	.95	.90 (.75-.95)	19±37	.95	.91 (.73-.95)
**Freesurfer LR BPV**						
SPM LR	-72±32	.96	.92 (.82-.97)	-69±34	.96	.93 (.80-.97)
**CSF LR ICV**						
FSL LR	-209±43*	.96	.92 (.63-.97)	-191±39*	.95	.91 (.63-.98)
FreeSurfer LR	-227±34*	.98	.96 (.87-.99)	-214±31*	.97	.94 (.81-.98)
SPM LR	-17±45	.94	.88 (.62-.94)	6±46	.94	.88 (.64-.94)
**CSF HR ICV**						
FSL LR	-191±41*	.96	.92 (.70-.96)	-191±39*	.96	.92 (.68-.97)
FreeSurfer LR	-209±27*	.99	.97 (.90-.99)	-214±31*	.98	.97 (.88-.99)
SPM LR	1±47	.94	.89 (.66-.93)	6±46	.94	.89 (.67-.93)
**FSL LR ICV**						
FreeSurfer LR	-18±39	.97	.93 (.83-.96)	-23±36	.97	.95 (.86-.98)
SPM LR	192±30*	.99	.97 (.90-.99)	197±31*	.98	.97 (.84-.99)
**Freesurfer LR ICV**						
SPM LR	210±58*	.93	.87 (.57-.92)	220±56*	.94	.89 (.70-.94)

CSF LR = CSF low resolution MRI scan; CSF HR = CSF high resolution MRI scan.

* $p < 0.05$
