# Supplementary material for: Fast CSF MRI for brain segmentation; Cross-validation by comparison with 3D T1-based brain segmentation methods
Source: PLoS One. 2018 Apr 19;13(4):e0196119. doi: 10.1371/journal.pone.0196119 (PMC5908081; doi:10.1371/journal.pone.0196119)
Supplement: S2 Table — (PDF) [file pone.0196119.s009.pdf]

	Scan 1			Scan 2		
Model[a]	Intercept (95% CI)	Slope (*B*) (95% CI)	R^2 (90% CI)	Intercept (95% CI)	Slope (*B*) (95% CI)	R^2 (90% CI)
**CSF LR BPV**						
FSL HR	54 (-238-345)	.98 (.74-1.21)	.92 (.77-.97)	108 (-246-462)	.94 (.65-1.23)	.88 (.64-.96)
FreeSurfer HR	91 (-85-267)	1.00 (.85-1.15)	.97 (.88-.99)	80 (-196-357)	1.01 (.78-1.25)	.92 (.75-.97)
SPM HR	30 (-268-327)	1.01 (.76-1.25)	.92 (.75-.96)	45 (-317-408)	1.00 (.70-1.30)	.88 (.60-.96)
**CSF HR BPV**						
FSL HR	37 (-302-375)	1.02 (.75-1.30)	.90 (.70-.97)	81 (-250-412)	.99 (.72-1.26)	.90 (.75-.97)
FreeSurfer HR	73 (-145-290)	1.05 (.86-1.24)	.95 (.82-.99)	39 (-146-225)	1.08 (.92-1.24)	.97 (.88-.99)
SPM HR	13 (-335-360)	1.06 (.77-1.34)	.90 (.65-.96)	12 (-318-342)	1.06 (.79-1.33)	.91 (.72-.96)
**FSL HR BPV**						
FreeSurfer HR	84 (-70-238)	.99 (.85-1.12)	.97 (.95-.99)	23 (-156-201)	1.04 (.88-1.19)	.97 (.92-.99)
SPM HR			.95 (.85-.98)			.95 (.86-.97)
**Freesurfer HR BPV**						
SPM	-70 (-267-128)	1.01 (.85-1.18)	.96 (.91-.98)	-39 (-235-157)	.99 (.83-1.15)	.96 (.91-.98)
**CSF LR ICV**						
FSL HR	63 (-413-539)	.89 (.59-1.18)	.86 (.44-.99)	127 (-332-586)	.85 (.56-1.14)	.85 (.47-.99)
FreeSurfer HR	783 (448-1117)	.45 (.24-.66)	.75 (.49-.90)	795 (456-1133)	.44 (.22-.65)	.74 (.52-.88)
SPM HR	131 (-310-571)	.89 (.60-1.18)	.86 (.48-.94)	200 (-260-660)	.85 (.54-1.15)	.84 (.42-.92)
**CSF HR ICV**						
FSL HR	-11 (-481-460)	.94 (.65-1.24)	.87 (.49-.98)	51 (-397-500)	.91 (.63-1.19)	.87(.53-.98)
FreeSurfer HR	787 (402-1173)	.46 (.21-.70)	.70 (.38-.88)	798 (410-1186)	.45 (.20-.69)	.69 (.40-.87)
SPM HR	64 (-375-502)	.95 (.66-1.24)	.88 (.53-.93)	125 (-320-571)	.91 (.61-1.20)	.86 (.53-.92)
**FSL HR ICV**						
FreeSurfer HR	877 (518-1236)	.46 (.24-.69)	.73 (.49-.87)	847 (480-1215)	.48 (.25-.71)	.74 (.48-.88)
SPM HR	144 (-205-493)	.96 (.73-1.19)	.92 (.75-.97)	142 (-229-514)	.96 (.71-1.20)	.91 (.73-.96)
**Freesurfer HR ICV**						
SPM HR	-764 (-2048-519)	1.53 (.69-2.38)	.69 (.41-.86)	-681 (-1967-604)	1.48 (.64-2.33)	.67 (.33-.86)

[a]Dependent variable printed in bold
