# Supplementary material for: Fast CSF MRI for brain segmentation; Cross-validation by comparison with 3D T1-based brain segmentation methods
Source: PLoS One. 2018 Apr 19;13(4):e0196119. doi: 10.1371/journal.pone.0196119 (PMC5908081; doi:10.1371/journal.pone.0196119)
Supplement: S3 Table — (PDF) [file pone.0196119.s010.pdf]

	Scan 1			Scan 2		
Model[a]	Intercept (95% CI)	Slope (*B*) (95% CI)	R^2 (90% CI)	Intercept (95% CI)	Slope (*B*) (95% CI)	R^2 (90% CI)
**CSF LR BPV**						
FSL LR	4 (-267-275)	1.00 (.78-1.21)	.93 (.84-.97)	-5 (-388-377)	1.00 (.70-1.31)	.88 (.62-.96)
FreeSurfer LR	101 (-99-302)	.98 (.81-1.15)	.96 (.82-.99)	24 (-300-348)	1.05 (.78-1.33)	.91 (.64-.98)
SPM LR	121 (-181-423)	.91 (.67-1.15)	.90 (.76-.95)	151 (-174-477)	.89 (.63-1.16)	.89 (.65-.94)
**CSF HR BPV**						
FSL LR	-11 (-344-321)	1.04 (.78-1.30)	.91 (.77-.96)	-52 (-372-268)	1.07 (.82-1.32)	.92 (.76 -.97)
FreeSurfer LR	88 (-168-345)	1.03 (.81-1.25)	.94 (.74-.99)	-19 (-270-231)	1.12 (.91-1.34)	.95 (.79 -.99)
SPM LR	107 (-238-453)	.96 (.68-1.23)	.89 (.68-.95)	133 (-184-450)	.94 (.68-1.19)	.90 (.76-.96)
**FSL LR BPV**						
FreeSurfer LR	135 (-54-325)	.96 (.80-1.12)	.96 (.88-.98)	63 (-95-222)	1.02 (.89-1.16)	.97 (.92-.99)
SPM LR	158 (-141-458)	.88 (.64-1.12)	.90 (.75-.95)	212 (-62-486)	.84 (.62-1.06)	.91 (.73-.95)
**FreeSurfer LR BPV**						
SPM	33 (-235-302)	.92 (.70-1.13)	.92 (.82-.97)	146 (-84-376)	.83 (.64-1.01)	.93 (.80-.97)
**CSF LR ICV**						
FSL LR	30 (-330-390)	.86 (.65-1.07)	.92 (.63-.97)	7 (-383-396)	.87 (.64-1.10)	.91 (.63-.98)
FreeSurfer LR	32 (-197-261)	.85 (.71-.98)	.96 (.87-.99)	76 (-218-370)	.82 (.65-.99)	.94 (.81-.98)
SPM LR	-1 (-444-442)	.99 (.70-1.28)	.88 (.62-.94)	-2 (-453-449)	.99 (.69-1.29)	.88 (.64-.94)
**CSF HR ICV**						
FSL LR	-34 (-404-335)	.91 (.69-1.13)	.92 (.70-.96)	-72 (-447-303)	.93 (.71-1.15)	.92 (.68-.97)
FreeSurfer LR	-36 (-241-169)	.90 (.78-1.02)	.97 (.90-.99)	-6 (-236-223)	.88 (.75-1.01)	.97 (.88-.99)
SPM LR	-67 (-525-392)	1.05 (.74-1.35)	.89 (.66-.93)	-78 (-532-377)	1.06 (.75-1.36)	.89 (.67-.93)
**FSL LR ICV**						
FreeSurfer LR	102 (-248-452)	.93 (.73-1.13)	.93 (.83-.96)	151 (-147-448)	.90 (.73-1.07)	.95 (.86-.98)
SPM LR	-46 (-276-185)	1.16 (1.01-1.31)	.97 (.90-.99)	-7 (-267-253)	1.14 (.96-1.31)	.97 (.84-.99)
**FreeSurfer LR ICV**						
SPM LR	8 (-538-555)	1.13 (.77-1.50)	.87 (.57-.92)	-48 (-562-467)	1.18 (.84-1.52)	.89 (.70-.94)

[a]Dependent variable printed in bold
